# l-Asparaginase from *Streptomyces griseus* NIOT-VKMA29: optimization of process variables using factorial designs and molecular characterization of l-asparaginase gene

**DOI:** 10.1038/srep12404

**Published:** 2015-07-24

**Authors:** Balakrishnan Meena, Lawrance Anburajan, Thadikamala Sathish, Rangamaran Vijaya Raghavan, Gopal Dharani, Nambali Valsalan Vinithkumar, Ramalingam Kirubagaran

**Affiliations:** 1 Andaman and Nicobar Centre for Ocean Science and Technology, Earth System Sciences Organization-National Institute of Ocean Technology (ESSO-NIOT), Port Blair-744103, Andaman and Nicobar Islands, India; 2 Marine Biotechnology Division, Ocean Science and Technology for Islands Group, ESSO-NIOT, Ministry of Earth Sciences, Government of India, Chennai-600100, India

## Abstract

Marine actinobacteria are known to be a rich source for novel metabolites with diverse biological activities. In this study, a potential extracellular L-asparaginase was characterised from the *Streptomyces griseus* NIOT-VKMA29. Box-Behnken based optimization was used to determine the culture medium components to enhance the L-asparaginase production. pH, starch, yeast extract and L-asparagine has a direct correlation for enzyme production with a maximum yield of 56.78 IU mL^−1^. A verification experiment was performed to validate the experiment and more than 99% validity was established. L-Asparaginase biosynthesis gene (*ans*A) from *Streptomyces griseus* NIOT-VKMA29 was heterologously expressed in *Escherichia coli* M15 and the enzyme production was increased threefold (123 IU mL^−1^) over the native strain. The *ans*A gene sequences reported in this study encloses several base substitutions with that of reported sequences in GenBank, resulting in altered amino acid sequences of the translated protein.

l-Asparaginase is an amidohydrolase which catalyses the hydrolysis of amino acid asparagine into aspartic acid and ammonia. Neoplastic cells cannot synthesize l-asparagine in the absence of l-asparagine synthase; therefore, they should be solely dependent on the circulating sources for l-asparaginase. The commonest therapeutic practice is to inject the l-asparaginase intravenously to decrease concentration of l-asparagine in the blood, thereby selectively affecting the neoplastic cells^1^. The asparaginase from *Escherichia coli* and *Erwinia carotovora* are currently being used in the treatment of acute lymphoblastic leukaemia. However, the prolonged administration of l-asparaginase, leads to anaphylactic shock in humans. Therefore, there is a continuing need to screen novel organisms that have the potential to produce high levels of l-asparaginase^2^.

Among the actinobacteria, several *Streptomyces* sp., such as *S*. *karnatakensis*, *S*. *venuzuzlae*, *S*. *longsporusflavus*, *S*. *gulbargensis* and marine *Streptomyces* sp. PDK2 have been explored for l-asparaginase production^3^. Extracellular asparaginases are more advantagious than intracellular ones as they can be produced in abundance in the culture broth under normal conditions and can be purified economically^4^. The Andaman coast of India is an outsized diverse and unexploited ecosystem, which can be explored for novel actinobacteria with effective bioactive molecules^5^. Marine actinobacteria with high alkalotolerent and halotolerant characteristics from Andaman and Nicobar (A & N) Islands and their pharmaceutical and industrial importance has already been reported by Meena *et al.*^5^. Microorganisms from these unexplored environments are expected to have proteins with features different from the organisms from other environments. In this study, an attempt was made to optimize the l-asparaginase production with various medium components using Box-Behnken experimental design and for overexpression of the l-asparaginase biosynthesis gene (*ans*A) from *S*. *griseus* NIOT-VKMA29.

## Methods

### Actinobacterial isolate and growth conditions. The actinobacterial strain,

NIOT-VKMA29 was isolated from the marine sediments of Phoenix Bay in Port Blair, A & N Islands, India and was grown aerobically in the Starch Casein Agar (SCA) supplemented with nalidixic acid (25 μg mL^−1^, Hi-Media, India) to inhibit the fast growing Gram-negative bacteria.

### Identification of the potential actinobacteria

Morphological, biochemical, cultivatory and physiological characterization of the potential isolate was performed as recommended in the International Streptomyces Project (ISP) described by Shirling and Gottileb^6^. Microscopic examination was performed by cover slip culture and cellophane method. Formation of aerial, substrate mycelium and spore arrangements on mycelium were monitored under a phase contrast microscope (Nikon Eclipse E600, USA) at 100× magnification and a scanning electron microscopy (TESCAN VEGA3, Czech Republic). Genomic DNA of actinobacterial strain NIOT-VKMA29 was isolated by following the modified procedure of Kutchma *et al.*^7^. Amplification of 16S rRNA was performed using universal primers 16Sf (5′ AGAGTTTGATCCTGGCTCAG 3′) and 16Sr (5′ GGTTACCTTGTTACGACTT 3′). Final volume of PCR was 25 μL, which comprised *Taq* buffer (1×), dNTP’s (200 μM) (MBI Fermentas, USA), forward and reverse primer (0.5 μM), MgCl_2_ (1.0 mM), *Taq* DNA polymerase (1.25 U) (MBI Fermentas), template (1 μL) and for the rest with autoclaved Milli Q water. PCR was performed in the initial denaturation at 98 °C for 3 min, followed by 30 cycles of reaction with denaturation at 94 °C for 1 min; annealing at 53 °C for 1 min; extension at 72 °C; and final extension at 72 °C for 10 min. PCR amplicon was analyzed on 1.5% agarose gel along with DNA molecular weight marker (MBI Fermentas). Positive amplicon as judged by the size were purified using QIAquick PCR purification kit (Qiagen, Germany) and sequenced on an ABI PRISM 377 genetic analyzer (Applied Biosystems, USA).

16S rRNA sequences of the potential strain were aligned manually in the GenBank database with BLAST^8^ and the sequences with 98–100% homology were considered for molecular taxonomy analysis. Multiple alignments of 16S rRNA sequences in this study and the sequences in GenBank database were performed with the CLUSTAL X program^9^. Phylogenetic trees were constructed by using the neighbor-joining and maximum-parsimony methods in Molecular Evolutionary Genetic Analysis (MEGA version 6.0)^10^ and bootstrap values based on 1,000 replication^11^.

### Primary screening and submerged fermentation

Primary screening for l-asparaginase production from *S. griseus* NIOT-VKMA29 was carried out in asparagine dextrose salt medium containing 1.0% l-asparagine (w**/**v), 0.2% dextrose (w**/**v), 0.1% K_2_HPO_4_ (w**/**v), 0.05% MgSO_4_.7H_2_O (w**/**v), 1.5% agar (w**/**v) supplemented with 0.009% phenol red (v**/**v) as an indicator^12^. Production of l-asparaginase was performed by shake flask method and the medium with culture and control was incubated at 30 °C for 10 days. l-Asparaginase production and the biomass were monitored every 24 h. Cell growth was expressed in dry weight of biomass^3^.

### Determination of l-asparaginase and l-glutaminase activity

l-Asparaginase activity in the culture filtrates of *S. griseus* NIOT-VKMA29 was determined according to the method of Imada *et al.*^13^. The rate of hydrolysis of l-asparagine was determined by measuring the ammonia released using Nessler’s reagent. Protein content in the culture filtrate was estimated by the standard protocol of Lowry *et al.*^14^ using bovine serum albumin as standard. Asparaginase activity was calculated and expressed as units/mg protein.

l-Glutaminase assay was carried out as described previously by Imada *et al.*^13^ using 189 mM l-glutamine as substrate in 50 mM Tris buffer (pH 8.6) and the absorbance was documented at 436 nm. One International Unit (IU) of l-glutaminase activity is defined as the amount of enzyme required to release 1 μmole of ammonia per mL min^−1^ at pH 8.6 and 37 °C.

### Standardization of culture conditions for l-asparaginase production

One hundred millilitre of production medium was inoculated with *S*. *griseus* NIOT-VKMA29 and incubated at various pH (5, 6, 7, 8, 9 and 10) and temperature (20, 25, 30, 35, 37, 40, 45 and 50 °C). The pH at which maximum activity occurs is considered the optimum pH and the temperature at which maximum yield of enzyme observed is considered the optimum temperature^3^.

The effects of different carbon (1%) and nitrogen (1%) sources on cell growth and l-asparaginase production was studied in the basal medium containing K_2_HPO_4_ (0.05%) and MgSO4.7H2O (0.01%) at pH 8. The effects of different concentrations of l-asparagine (0.5–2.5%) were examined for l-asparaginase production under induced conditions to check for stimulation of l-asparaginase production by following the procedure of Sudhir *et al.*^4^.

### Optimization of l-asparaginase production by Box-Behnken design

Based on the preliminary studies, 4 parameters, viz., concentrations of l-asparagine (A), yeast extract (Y), starch (S) and pH (P) were identified as factors with a strong influence on l-asparaginase production by *S*. *griseus* NIOT-VKMA29. A Box-Behnken design (BBD) with a total of 27 experiments consisting of 24 factorial points (−1 and +1) and 3 central points (0, 0) were employed to understand the influence of the 4 selected parameters on l-asparaginase production. Table 1 shows the experimental plan and the levels of independent variables. All variable levels *X*_*i*_ were coded as *x*_*i*_ according to the following equation, so that *X*_*0*_ corresponded to the central value:


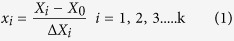


Where *x*_*i*_ = dimensionless value of an independent variable, *X*_*i*_ = real value of an independent variable, *X*_*0*_ = real value of an independent variable at the central point, and Δ*X*_*i*_ = step change.

The obtained data was fitted by a second-order model to correlate the experimental

l-asparaginase activity to the studied variables. The general form of the second-degree polynomial equation is,


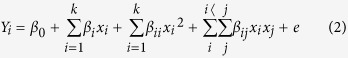


Where *Y*_*i*_ = predicted response (l-asparaginase activity), *xi* & *xj* = input variables that influence the l-asparaginase production, *β*_*0*_ = offset term, *β*_*i*_ = *i*^th^ linear coefficient, *β*_*ii*_ = *i*^th^ quadratic coefficient, and *e* = error.

The analysis of model was carried out in the form of analysis of variance (ANOVA). The R^2^ statistic indicates the percentage of the variability of optimization parameters that were explained by the model. Three-dimensional surface plots were drawn to illustrate the main and interactive effects of the selected variables on l-asparaginase production. The design was analyzed and the coefficients of the model were tested for their significance by linear regression analysis using MATLAB 6.0. All experiments were conducted in triplicate and mean values were used for analysis.

### PCR amplification of l-asparaginase biosynthesis gene

l-Asparaginase biosynthesis gene (*ans*A) of *S*. *griseus* NIOT-VKMA29 was PCR amplified by using gene specific primers designed using Primer3 programme available at http://frodo.wi.mit.edu/primer3 (Table 2). PCR was performed in 50 μL of reaction mixture, which contained 50 ng of genomic DNA, 0.5 μM of each primer, 200 μM each of dNTP (MBI Fermentas), 1.25 U of *Pfu* DNA polymerase (MBI Fermentas), 1× *Pfu* buffer; 2.5 mM of MgSO_4_ and for the rest autoclaved Milli Q water. Amplification was performed in a Master cycler (Eppendorf, Germany) with the following conditions: initial denaturation at 94 °C for 3 min, followed by 30 repeated cycles of 94 °C for 30 s, 52 °C for 1 min, 72 °C for 2 min and final extension at 72 °C for 10 min. PCR amplicons were analyzed on 1.5% agarose gel along with DNA molecular weight marker (MBI Fermentas) and documented in gel documentation system (UVP BioSpectrum Imaging system, USA).

### Cloning and sequencing

PCR amplicon of *ans*A was purified by MinElute Gel purification Kit (Qiagen, Germany) and cloned into pTZ57R/T (MBI Fermentas), according to the manufacturer’s instructions. pTZ57R/T-*ans*A construct was transformed into competent *E*. *coli* JM109 (*recA*1, *endA*1, *gyrA*96, *thi*-1, *hsdR*17 (rK–mk+), e14–(*mcrA*−), *supE*44, *relA*1, Δ(*lac-proAB*)/F’ (*traD*36, *proAB*+, *lac I*q, *lacZ* ΔM15) and plated on Luria Bertani (LB) agar containing ampicillin (100 μg mL^−1^), isopropyl-β-D-thiogalactoside (IPTG, 50 μM) and X-gal (80 μg mL^−1^) and incubated overnight at 37 °C. White colonies were selected for PCR amplification with vector primers M13f-M13r (MBI Fermentas) and the clones with correct insert as judged by size were sequenced on an ABI PRISM 377 genetic analyzer (Applied Biosystems Inc., USA).

### *In silico* sequence analysis

The obtained nucleotide sequences were compared with database sequences using BLAST provided by NCBI (http://www.ncbi.nlm.nih.gov) and multiple sequence alignment of the blast hits were carried out using Clustal Omega. The output alignments were imported into the GeneDoc program (http://www.psc.edu/biomed/genedoc/) and the version 7.05 of BioEdit program (www.mbio.ncsu.edu/BioEdit/) to calculate the percent identities among the nucleotide and amino acid sequences. The phylogenetic analyses were carried out using MEGA 6.0 program. The molecular masses and theoretical pI values of the polypeptides were predicted using the ProtParam tool (http://www.expasy.org/tools/protparam.html). The sequence generated in this study was deposited in the GenBank database under the accession number KF724083.

### Heterologous expression of l-asparaginase biosynthesis gene

The recombinant plasmid pTZ57R/T-*ans*A cassette was double digested with *Eco*RI and *Bam*HI (MBI Fermentas) and purified by the MinElute gel purification kit. The purified *ans*A gene was religated into pQE30 (Qiagen, Germany), which had previously been digested and purified. The resulting recombinant expression vector pQE30-*ans*A cassette was transformed into the competent *E*. *coli* M15. The overexpression of the l-asparaginase gene in the host cell was performed by following the methodology provided in the QIAexpress Type IV Kit (Qiagen). A single colony of the recombinant clone was inoculated into 10 mL of LB broth containing 100 μg mL^−1^ ampicillin and 25 μg mL^−1^ kanamycin and incubated overnight at 37 °C. Approximately 5.0 mL of the culture was transferred into 100 mL LB broth containing 100 μg mL^−1^ ampicillin and 25 μg mL^−1^ kanamycin and incubated at 37 °C until the OD_600_ value was reached 0.6–0.7. IPTG was then added to the culture broth at the final concentration of 1 mM, and was continuously incubated at 37 °C for 4 h. The induced bacterial cells were harvested by centrifugation and resuspended in 1 × SDS-PAGE sample buffer and lysed in boiling water bath for 3 min. The cells were centrifuged at 4,000 × *g* for 20 min and the supernatant was checked for soluble proteins to study the expression. The expression of target protein was analyzed by sodium dodecyl sulfate-polyacrylamide gel electrophoresis (SDS-PAGE) as described by Laemmli,^15^. The molecular mass was estimated with the protein ladder (Sigma, USA).

## Results and Discussion

### Identification of the potent strain

**Figure 1:**
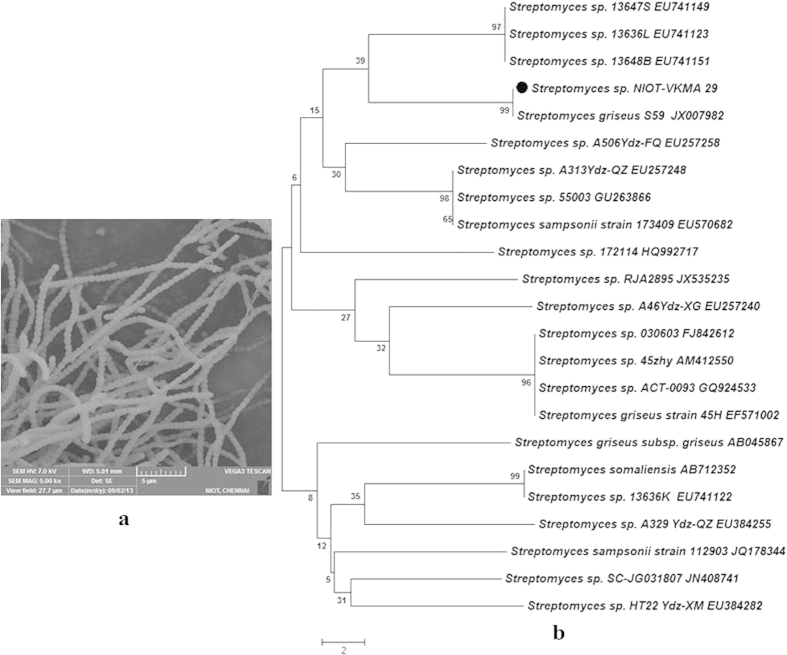
(**a**) Scanning electron microscopic image of *S. griseus* NIOT-VKMA29 (**b**) Phylogenetic tree based on 16S rRNA sequences using neighbor-joining method for *S. griseus* NIOT-VKMA29. Branch distances represent nucleotide substitution rate and scale bar represents the number of changes per nucleotide position.

The actinobacterial strain, NIOT-VKMA29 is a Gram-positive, non-acid fast, non-motile, aerobic, and filamentous organism, with very long rods, spores on aerial mycelium are rectiflexible chains as observed by cover-slip method and evaluated by phase contrast microscope (Nikon Eclipse E600, USA) and scanning electron microscopy (TESCAN VEGA3, Czech Republic) (Fig. 1a). The 16S rRNA sequences (1,427 bp) generated in this study were deposited in GenBank under the accession number KF031012. Upon analysis with the BLAST program and phylogenetic analysis, it was established that the deduced nucleotide sequences of NIOT-VKMA29 was highly homologous (100%) with the reported 16S rRNA sequences of *S. griseus* (GenBank accession no. JX007982) (Fig. 1b). Based on the morphological and biochemical characteristics (Table 3) and the phylogenetic analysis, the isolate NIOT-VKMA29 was identified as *S*. *griseus*.

### Standardization of culture conditions for l-asparaginase production

**Figure 2:**
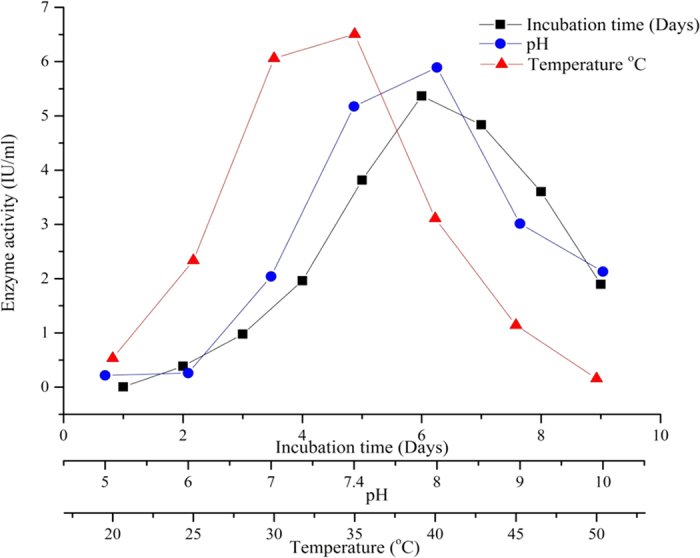
Effect of batch time, pH and temperature (°C) on enzyme activity.

The potent strain was subjected to submerged fermentation to determine the production of l-asparaginase. The production medium was inoculated with *S. griseus* NIOT-VKMA29 and the enzyme activity was analyzed every 24 h of incubation. Production of l-asparaginase started after 24 h of incubation and reached the maximum after 144 h of incubation (6^th^ day 5.36 IU mL^−1^). When the production was carried out in ADS broth at pH 7.4 and temperature 37 °C, the l-asparaginase activity reached 5.36 IU mL^−1^ (Fig. 2) with merely 0.18 IU mL^−1^ of glutaminase activity. Amena *et al.*^2^ and Mostafa and Salama^16^, reported the maximum l-asparaginase production on 6^th^ day in *Streptomyces gulbargensis* and *Streptomyces collinus*, respectively.

A study of initial pH levels (5–10) on the production of l-asparaginase by *S. griseus* NIOT-VKMA29 indicated optimum enzyme production at pH 8 (5.97 IU mL^−1^). It was found that the strain grows well at an alkali pH of 8 and optimum temperature for enzyme production ranged from 30–35 °C in the optimized production medium. Narayana *et al.*^3^ also revealed maximum l-asparaginase production at 35 °C by *S. albidoflavus*. Extreme pH and temperature did not favor cell growth as well as production of l-asparaginase by *S. griseus* NIOT-VKMA29. The l-asparaginase production was maximum by *S. griseus* NIOT-VKMA29 in the production medium with optimized initial pH 8 for 144 h at 35 °C (Fig. 2).

**Figure 3:**
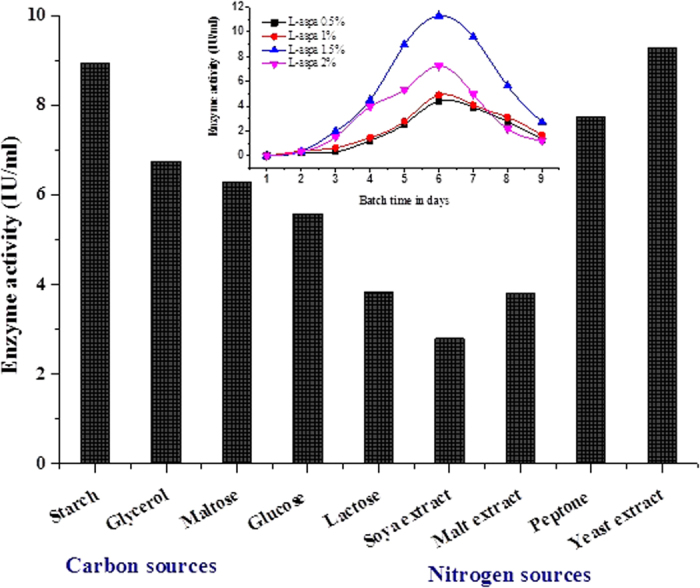
Effect of carbon, nitrogen and l-asparagine concentration on enzyme production.

Different carbon sources like starch, glycerol, maltose, glucose and lactose were amended in the production medium to determine their impact on l-asparaginase production. As compared to various carbon sources tested, l-asparaginase production was high (8.93 IU mL^−1^) in the production medium containing starch (1%) as the carbon source (Fig. 3). The production of l-asparaginase was marginally favourable with low cost substrates viz., starch, glycerol and maltose and there is no significant effect with glucose and lactose. Abdel-Fatah^17^ also reported starch as the best carbon source for l-asparaginase production in *Streptomyces longsporusflavus*. The effect of nitrogen compounds on the production of l-asparaginase by strain *S. griseus* NIOT-VKMA29 was studied using various nitrogen sources such as soya peptone, malt extract, peptone and yeast extract. l-Asparaginase production by *S. griseus* NIOT-VKMA29 varied with the different nitrogen sources (Fig. 3). Among them, culture medium amended with yeast extract favoured maximum l-asparaginase production (9.27 IU mL^−1^) followed by peptone. The final pH of fermentation broth consisting of starch, glycerol and maltose developed alkaline conditions, whereas in medium amended with other carbon sources it developed acidic, which may have led to the decline in productivity of l-asparaginase. The acidity of the fermentation medium could inhibit the production of l-asparaginase^18^, and because of this nature, glucose is reported to be the repressor for l-asparaginase production^19^. Observations are recorded on the enhancement of l-asparaginase synthesis by different concentrations of l-asparagine in the culture medium. This study was carried out based on the earlier reports that a synthetic medium with l-asparagine as nitrogen source stimulated more enzyme production than biologically by *Streptomyces* sp.^16^. Thus, medium containing different concentrations (0.5–2.5%) of l-asparagine were used and the maximum l-asparaginase activity was found to be 11.27 IU mL^−1^ at 1.5% of l-asparagine (Fig. 3).

### Optimization of l-asparaginase production by Box-Behnken design

The l-asparaginase activity corresponding to the Box-Behnken experimental run was used as a response for analysis. Table 3 shows that the production of l-asparaginase varies from 20.18 to 56.78 (IU mL^−1^), indicating the dominant role of selected variables and their concentrations on enzyme production by *S. griseus* NIOT-VKMA29. The low percentage of variation between the observed and predicted values indicates the accuracy of the experiment. From the results it was clear that only the 3^rd^ run had an error of 11.60%; in all the other runs it was nearer to 5%, which shows the experiments were conducted with precision.

**Figure 4:**
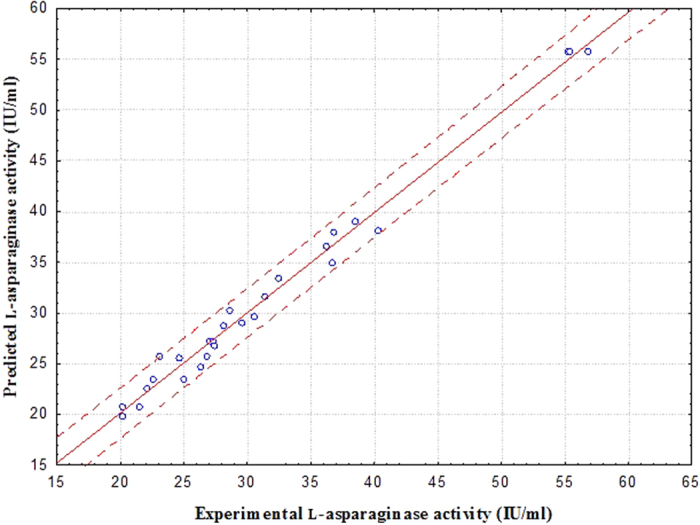
Correlation between the experimental and predicted l-asparaginase activity.

A multiple regression analysis was performed on the obtained data and the accuracy of the data was tested by the regression coefficient (R^2^). The regression coefficient (R^2^) was 0.9878, indicating that only 1.22% of the variability in the response could not be explained by the model. The high value of adjusted R^2^ (0.9737) suggested a higher significance of the model. The correlation plot (Fig. 4), obtained a high adjusted R^2^ value of 0.9737, also indicated that the obtained regression model given a good explanation of the relationship between the independent and response variables. In the present experiment, the coefficient of variance (CV) was 5.32%, which implies good precision and reliability. The application of response surface methodology (RSM) yielded the following regression equation, which is an empirical relationship between the selected parameters and l-asparaginase production.


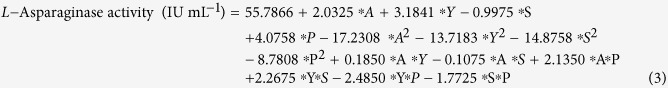


The coefficients were selected based on their corresponding t, F, and p-values (Table 4). The overall p-value of the model is <0.0001 and the F-value is 69.89 (model F-value > p-value), implying that the model is significant. Further, to confirm the acceptance of model, a precision test was performed, which measures the signal to noise ratio. The precision value greater than 4 is considered adequate. In the present experiment, a ratio of 28.789 was observed, indicating an adequate signal. The correlation coefficient, model p, and F values, and an adequate precision value suggest that the proposed model could be used to navigate the design space.

Coefficients which have a low p-value and high F-value are considered as significant terms. Based on this linear term of starch (S), the interaction terms of l-asparagine with yeast extract, starch (A*Y, A*S) and starch *vs.* pH (S*P) are insignificant terms. The square term of l-asparagine had the highest effect indicating that it was the most important component for l-asparaginase production. The square terms of l-asparagine, starch and yeast extract have a higher effect value than the linear terms, indicating that these variables have more influence on the enzyme production; any alteration in these levels could influence the production in a significant manner.

**Figure 5:**
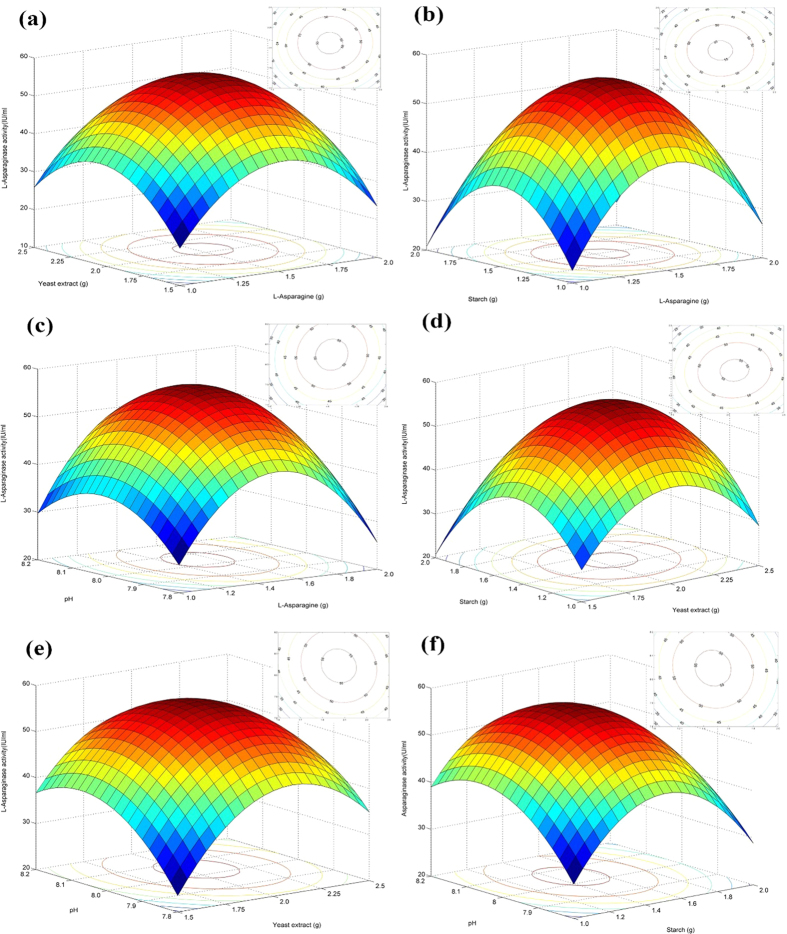
Surface and contour plots of the selected parameters interaction: (**a**) l-asparagine with yeast extract (**b**) l-asparagine with starch (**c**) l-asparagine with pH (**d**) yeast extract with starch (**e**) yeast extract with pH (**f**) starch with pH.

The regression equation (Eq. 3) developed here was used to generate 3D and 2D surface & contour plots respectively. Using the drawn surface and contour plots’ interactions, selected parameters at different conditions were evaluated. All contours were circular or elliptical in nature, indicating’s that all selected parameters were independent of each other. Figure 5a,c depicts the interaction of l-asparagine with other selected variables; it was observed that l-asparagine concentration was independent of the carbon and nitrogen sources. l-Asparagine at the concentration range of 1.4–1.6% was optimum for effective l-asparaginase production by *S. griseus* NIOT-VKMA29. Figure 5a,d,e represents the interaction of yeast extract with other parameters. In Fig. 5e, the contour is slightly inclined towards pH indicating that yeast extract concentration has a slight influence on pH of the medium. From Fig. 5a,d,e it can be seen that yeast extract at a concentration range of 1.9–2.1% is optimum. Figure 5d represents the interaction of carbon and nitrogen sources; this contour is elliptical in nature indicating that both were independent of each other. Figure 5e,f indicates that yeast extract and starch have a minimal influence on the culture pH.

A numerical method given by Myers and Montgomery,^20^ was used to solve the regression equation. The optimal values of parameters were as follows: l-asparagine concentration 1.53%, yeast extract concentration 2.04%, starch concentration 1.48% and pH 8.04, with corresponding l-asparaginase production at 56.50 (IU mL^−1^). On conducting the experiments under these conditions, 56.81 IU/mL of enzyme was obtained. Liu and Zajic,^21^ also found that yeast extract stimulates the production of l-asparaginase in *Erwinia aroideae*. Yeast extract is essential for cell growth and l-asparaginase production but higher concentrations inhibit the production in *Streptomyces albidoflavus*^3^. Sahu *et al.*^22^ reported that the *Streptomyces* sp. such as *S*. *aureofasciculus* LA-2, *S. canus* LA-29 and *S*. *olivoviridis* LA-35 exhibited optimum growth and l-asparaginase production at pH range of 7–8.

### PCR amplification, molecular cloning and ***in silico*** analysis of l-asparaginase gene

**Figure 6:**
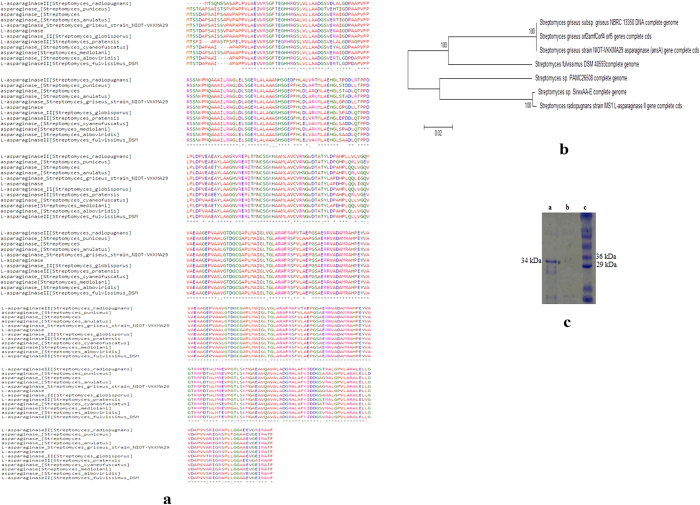
(**a**) Multiple sequence alignment of amino acid substitutions of l-asparaginase in *S. griseus* NIOT-VKMA29 with GenBank reports (**b**) Phylogenetic tree analysis using neighbour-joining algorithm with 1000 bootstrap replicates depicting the evolutionary relationship between l-asparaginase from other *Streptomyces* species. (**c**) SDS-PAGE analyses of the expressed *ans*A gene of *S. griseus* NIOT-VKMA29. Lane a, Total protein of the IPTG induced *ans*A gene cassette; Lane b, Total protein of the uinduced *ans*A gene cassette; Lane c, Protein molecular mass marker.

The asparaginase biosynthesis gene (*ans*A) of *S. griseus* NIOT-VKMA29 encodes polynucleotides of 987 bp, composed of 328 amino acids, with a molecular mass of 33721 Da., based on the *in silico* estimates (http://www.expasy.org/tools/protparam.html). The isoelectric point of the *ans*A protein was determined as 5.73. The asparaginase gene was cloned in pTZ57R/T and transformed into *E*. *coli* JM109. *In silico* sequence analysis revealed that the amino acid sequences of *ans*A gene shared a significant similarity to the l-asparaginase sequences from other *Streptomyces* species (Fig. 6a). Phylogenetic tree analysis also construes the higher level of homology between the sequences (Fig. 6b). The conserved domain (http://www.ncbi.nlm.nih.gov/Structure/cdd) and motif scan (http://myhits.isb-sib.ch/cgi-bin/motif_scan) analysis revealed that the l-asparaginase of *S. griseus* NIOT-VKMA29 belongs to l-Asparaginase II family and the amino acid sequences were significantly conserved among the previous reports from *Streptomyces* species.

### Functional characterization of l-asparaginase biosynthesis gene

The recombinant expression vector pQE30-*ans*A cassette was transformed into *E*. *coli* M15(pREP4). The expression of asparaginase biosynthetic gene was confirmed by Nesslerization assay. The enzyme activity in expressed cells was 123 IU mL^−1^, which was three times more than that of control cells. The asparaginase biosynthesis gene *ans*A have been functionally characterized in *Nocardiopsis alba*^23^. Expression of the asparaginase gene was analysed by SDS-PAGE electrophoresis. The lysates of induced cells revealed a clear expressed band with molecular mass of 34 kDa that correspond to *ans*A, which was not present in the uninduced cells (Fig. 6c). Molecular weight of the purified recombinant asparaginase was similar to the asparaginase reported from *S*. *albidoflavus*^3^.

## Conclusion

The production of l-asparaginase from *S. griseus* NIOT-VKMA29 was increased from 5.36 to 56.78 IU mL^−1^ in 6^th^ day after optimization using Box-Behnken design with the following conditions; pH 8.04, l-asparagine 1.53%, yeast extract 2.04% and starch 1.48%. Moreover, the engineered *E*. *coli* with l-asparaginase biosynthesis gene has the potential pharmaceutical and industrial application, since it can produce the glutaminase free anti-tumour enzyme at high rates and can avoid the complex down streaming process associated with conventional bioprocess.

## Additional Information

**How to cite this article**: Meena, B. *et al.* L-Asparaginase from *Streptomyces griseus* NIOT-VKMA29: optimization of process variables using factorial designs and molecular characterization of L-asparaginase gene. *Sci. Rep.* **5**, 12404; doi: 10.1038/srep12404 (2015).

## Figures and Tables

**Table 1:** Box-Behnken design along with experimental and predicted l-asparaginase activity (IU/mL).

Sl. No.	l-Asparagine		Yeast extract		Starch		pH		l-Asparaginase activity (IU/mL)		
	Coded	Real	Coded	Real	Coded	Real	Coded	Real	Experimental	Predicted	Error
01	−1	1.0	−1	1.5	0	1.5	0	8.0	20.18	19.81	0.37
02	1	2.0	−1	1.5	0	1.5	0	8.0	24.97	23.50	1.47
03	−1	1.0	1	2.5	0	1.5	0	8.0	23.12	25.80	−2.68
04	1	2.0	1	2.5	0	1.5	0	8.0	28.65	30.24	−1.59
05	0	1.5	0	2.0	−1	1.0	−1	7.8	27.07	27.28	−0.21
06	0	1.5	0	2.0	1	2.0	−1	7.8	28.12	28.83	−0.71
07	0	1.5	0	2.0	−1	1.0	1	8.2	38.47	38.98	−0.51
08	0	1.5	0	2.0	1	2.0	1	8.2	32.43	33.44	−1.01
09	0	1.5	0	2.0	0	1.5	0	8.0	55.23	55.79	−0.56
10	−1	1.0	0	2.0	0	1.5	−1	7.8	26.86	25.80	1.06
11	1	2.0	0	2.0	0	1.5	−1	7.8	24.58	25.60	−1.02
12	−1	1.0	0	2.0	0	1.5	1	8.2	30.55	29.68	0.87
13	1	2.0	0	2.0	0	1.5	1	8.2	36.81	38.02	−1.21
14	0	1.5	−1	1.5	−1	1.0	0	8.0	27.34	27.27	0.07
15	0	1.5	1	2.5	−1	1.0	0	8.0	29.57	29.11	0.46
16	0	1.5	−1	1.5	1	2.0	0	8.0	20.13	20.74	−0.61
17	0	1.5	1	2.5	1	2.0	0	8.0	31.43	31.65	−0.22
18	0	1.5	0	2.0	0	1.5	0	8.0	55.35	55.79	−0.44
19	−1	1.0	0	2.0	−1	1.0	0	8.0	22.14	22.54	−0.40
20	1	2.0	0	2.0	−1	1.0	0	8.0	27.40	26.82	0.58
21	−1	1.0	0	2.0	1	2.0	0	8.0	21.54	20.76	0.78
22	1	2.0	0	2.0	1	2.0	0	8.0	26.37	24.61	1.76
23	0	1.5	−1	1.5	0	1.5	−1	7.8	22.65	23.54	−0.89
24	0	1.5	1	2.5	0	1.5	−1	7.8	36.65	34.88	1.77
25	0	1.5	−1	1.5	0	1.5	1	8.2	36.26	36.66	−0.40
26	0	1.5	1	2.5	0	1.5	1	8.2	40.32	38.06	2.26
27	0	1.5	0	2.0	0	1.5	0	8.0	56.78	55.79	0.99

**Table 2:** Gene specific primers for l-asparaginase gene.

Gene	Primer sequences 5′-3′	Reference
*ans*A	ATGACGTCCACCGACGC	This study
	TCAGAATGTCGCGCGAAT	

**Table 3:** Phenotypic characteristics of selected actinobacteria.

Properties	*Streptomyces griseus*NIOT-VKMA29
Morphological characteristics	
Spore morphology	Chain
Colour of aerial mycelium	Green
Colour of substrate mycelium	Grey
Soluble Pigment	Greenish brown
Spore mass	Green
Biochemical characteristics	
Gram staining	+
Indole production	−
Methyl Red	+
Voges Proskauer	−
Citrate Utilization	+
H_2_S production	−
Nitrate reduction	+
Urease	+
Catalase	−
Oxidase	+
Melanin production	−
Starch hydrolysis	+
Haemolysis	+
Triple sugar iron	alk/alk
Survival at 50 °C	Moderate
Carbon source utilization	
Starch	+
Dextrose	−
Fructose	+
Maltose	+
Mannitol	+
pH	
5	+
6	+
7	+
8	+
9	+
10	+
11	+
NaCl tolerence (%)	
5	+
10	+
15	+
20	+
25	+
30	+

**Table 4:** ANOVA, main effects and coefficients.

Source	Effect	Coefficients	SS*	DF*	MS*	t-value	F-value	p-value
**Model**	–	–	–	–	–	–	69.89	<0.0001
**Intercept**	19.3828	55.7867				34.6784		0
**A**	4.0650	2.0325	49.5727	1	49.5727	4.199	17.6314	0.0012
**Y**	6.3683	3.1842	121.667	1	121.667	6.5782	43.2729	0
**S**	−1.9950	−0.9975	11.9401	1	11.9401	−2.0607	4.2467	0.0617
**P**	8.1517	4.0758	199.349	1	199.349	8.4203	70.9018	0
A*A	17.2308	−17.231	1583.48	1	1583.48	23.7316	563.1900	0
Y*Y	13.7183	−13.718	1003.69	1	1003.69	18.8939	356.9810	0
S*S	14.8758	−14.876	1180.22	1	1180.22	20.4881	419.7630	0
P*P	8.7808	−8.7808	411.216	1	411.216	12.0936	146.2560	0
A*Y	0.3700	0.1850	0.1369	1	0.13690	0.2207	0.0487	0.8291
A*S	−0.2150	−0.1075	0.0462	1	0.0462	−0.1282	0.0164	0.9001
A*P	4.2700	2.1350	18.2329	1	18.2329	2.5465	6.4848	0.0256
Y*S	4.5350	2.2675	20.5662	1	20.5662	2.7046	7.3147	0.0191
Y*P	−4.9700	−2.4850	24.7009	1	24.7009	−2.9640	8.7853	0.0118
S*P	−3.5450	−1.7725	12.5670	1	12.5670	−2.1142	4.4697	0.0561
**Residual**			33.7394	12	2.8116			
**Lack of fit**			32.2521	10	3.2252		4.3371	0.2018
**Error**			1.4872	2	0.7436			
**Total**			2784.69	26				

^*^SS–sum of squares, DF–degrees of freedom, MS–mean square.
